# Informed consent practice and associated factors among healthcare professionals in public hospitals of Southern Ethiopia, 2023: a mixed-method study

**DOI:** 10.1186/s12912-024-01748-9

**Published:** 2024-01-30

**Authors:** Getachew Nigussie Bolado, Bizuayehu Atnafu Ataro, Mulualem Gete Feleke, Christian Kebede Gadabo, Tamirat Ersino Kebamo, Worku Mimani Minuta

**Affiliations:** 1 Adult Health Nursing, School of Nursing, College of Health Science and Medicine, Wolaita Sodo University, Sodo, Ethiopia; 2 Pediatrics and Child Health Nursing, School of Nursing, College of Health Science and Medicine, Wolaita Sodo University, Sodo, Ethiopia; 3 Department of Medical Laboratory, School of Nursing, College of Health Science and Medicine, Wolaita Sodo University, Sodo, Ethiopia; 4 Department of Public Health, Jinka University, Jinka, Ethiopia

**Keywords:** Informed consent, Hospitals, Factors, Healthcare professionals

## Abstract

**Background:**

Patients may sign a consent form before the specific treatment is offered for a variety of reasons, including during an outpatient appointment. Healthcare professionals must obtain consent from patients or other legal persons before providing any treatment or performing any procedures. But, little attention has been given to the informed consent process in Ethiopia.

**Objective:**

To assess informed consent practice and associated factors among healthcare professionals in Wolaita Zone, Southern Ethiopia public hospitals from January, 2023.

**Methods:**

An institutional-based cross-sectional mixed-method study was conducted among 399 healthcare professionals. Simple random sampling and purposive sampling techniques were used to select healthcare professionals for quantitative and qualitative studies respectively. Data for both studies were collected using self-administered questionnaire and key informant interview respectively. EpiDataV4.6 and the Statistical Package for the Social Science was used for entry and analysis of quantitative data. OpenCode software was used for thematic analysis for qualitative data.

**Results:**

339 respondents were included in the study, with a response rate of 94.3%. The good practice of informed consent among the healthcare professionals is 53.1%. There was a significant association between the good practice of informed consent and being male [AOR: 0.003 (95% CI: 0.000–0.017)], working in a comprehensive specialized hospital [AOR: 4.775 (95% CI: 1.45–15.74)] and in-service training [AOR: 0.038 (95% CI: 0.013–0.114)].

**Conclusion and recommendations:**

More than half of healthcare professionals had good practices for informed consent. However, it is critical to plan and intervene various strategies with the goal of improving knowledge and attitude toward informed consent.

**Supplementary Information:**

The online version contains supplementary material available at 10.1186/s12912-024-01748-9.

## Introduction

The term “informed consent” refers to a person’s freely expressed decision to accept a medical treatment, protocol, or other intervention following receipt of exact and pertinent information about the intervention and available alternatives, as well as having a sufficient understanding of the risks and benefits of the proposed intervention that are pertinent to the person who would receive the treatment, procedure, or other intervention [1, 2]. It highlights the patient’s autonomy and can be considered one of the pillars of nursing intervention, along with competence, disclosure, knowledge, involuntariness, and agreement [3, 4].Informed consent is a flexible (not rigid) approach that gives patients the precise information they need to make informed decisions [5]. A discussion between a healthcare professional and the patient, who is given the choice to participate in the treatment options as well as an informed consent document, is the first step in the informed consent process [6, 7].

The concept of informed consent has been codified in Sects. 37 and 38 of the 1999 Constitution as a basic right in Nigeria, and it complies with other international standards that acknowledge the importance of informed consent in medical and scientific practice [8] In sub-Saharan African countries, informed consent is influenced by cultural background, family structure, socioeconomic status, religion, and education [9]. Surgical consent can help patients safeguard themselves from unwelcome procedures, maintain their autonomy, and uphold their moral and legal rights, and patient happiness is also largely influenced by informed consent, a legal aspect of the practice of healthcare; in fact, patient satisfaction is crucial for any medical professional [10].

Informed consent allegations, battering, injury to the patient, incorrect treatment and surgical site operations, poor outcome and prognosis of the procedure, and indemnity payments are all examples of cases involving medical malpractice where proper informed consent was not obtained. More than 30% of claims involving informed consent involved failing to disclose risks and negative effects of operation [11, 12]. The study conducted at Tikur Anbessa specialized hospital in Addis Ababa revealed that patients’ awareness about their rights was 76% [13]. Another cross-sectional mixed qualitative and quantitative study conducted among service providers and patients in Addis Ababa public hospitals showed that the standard of informed consent is not fully followed, and patients are not adequately informed about the risks, options, and complications associated with the procedure. The patient who was better educated understood the purpose and application of the surgical informed consent guideline [14]Patient anxiety, postoperative dissatisfaction, and patient safety incidents are all increased when patients lack awareness about surgical informed consent [15].

According to a study conducted at the Gondar University Comprehensive and Specialized Hospital, 63.91% of healthcare professionals had inadequate knowledge, and 51.3% had inadequate perceptions of the study’s focus on surgical informed consent [16]. Patients in specialized hospitals in Ethiopia are entitled to the following rights, including healthcare and respect as human beings, informed consent, a clean and safe environment, health promotion, the right to choose their healthcare provider and to participate in decision-making and be heard, as well as the right to file complaints [13, 14]. Another cross-sectional study in Italy and Uganda revealed that the practice of informed consent was 71% and 48.8%, respectively [17, 18].

There are many contributing factors affecting the practice of informed consent. Excessive workload, language barriers, medical terminologies, educational differences, cultural/traditional differences, patient comprehension, use of disclosed information, autonomy, insufficient time allocation, and the degree to which health care providers adhere to the minimal standards for disclosure all have an impact on the practice of proper informed consent [12, 19]Other challenges to informed consent implementation include poverty, a lack of educational advancement, lack of familiarity with autonomy and individual freedom of patient, and power imbalance [20, 21]. Similarly, a cross-sectional study conducted in among surgical patients attending public hospitals in Dessie city administration showed that educational status, rural residence, marital status, language variation, poor patient-physician relationship, and poor knowledge of surgical informed consent had significant effect on practice of informed consent [10]. The studies conducted in Nigist Eleni Mohammed Memorial Comprehensive Specialized Hospital and Padua Hospital revealed that language barrier, race, education, age, availability of policy/regulation and Padua Hospital revealed that language barrier, race, education, age, and total time of the informed consent process were predictors of informed consent practice, whereas, gender, marital status, anxiety, and reading level were not associated with informed consent practice [22, 23].

Two distinct issues with the implementation and practice of informed consent are faced by African nations. Patient illiteracy and patriarchal attitudes are two obstacles to implementing informed consent. According to patriarchal attitudes, a person cannot offer consent without the approval of a third party, such as a husband, community leader, or elder due to their significant social role. The reality of heterogeneous cultures, including multilingualism, presents additional difficulties for the practice of informed consent [20].

There are limited studies conducted in Ethiopia, and most of them were conducted on patients as study participants. However, no study has been conducted in the study area on healthcare professionals. By addressing the gaps in information about the predictors of informed consent implementation from the perspective of healthcare professionals, this study sheds light on the subject and offers valuable insights for hospital management and stakeholders. The intention is to provide a basis for informed decision-making and prompt action to improve the overall implementation of informed consent practices. To the best of our knowledge, almost all studies that have been done in the past on healthcare professionals in Ethiopia have only used a quantitative study design, which is insufficient to uncover factors related to the practice of informed consent. Because of this, our study adopted a mixed-method approach. On the other hand, there are additional variables that have not yet been researched in Ethiopia but may be associated with the practice of informed consent, such as the level of hospital and the salary scale for healthcare professionals. The impact of these variables on the practice of informed consent was examined in this study. Therefore, this study assessed the practice of informed consent and associated factors among healthcare professionals in public hospitals in Wolaita Zone, Southern Ethiopia. The findings of the study will help healthcare professionals, hospitals, healthcare sectors, policymakers, and future researchers provide information to plan and take appropriate actions to improve the practice of informed consent.

## Methods and materials

### Study setting, design and period

This institutional-based cross-sectional mixed-method study was carried out among healthcare professionals working in public hospitals in Wolaita Zone, Southern Ethiopia. Wolaita Zone is one of the zones found in the newly established South Ethiopian Peoples Region, located 327 km and 151 km away from the country’s capital, Addis Ababa, at a latitude of 6° 54’’ north and a longitude of 37° 45’’ east. Its capital city is called Sodo, which is also serving as the capital city of the new region. Based on the 2021 population projection conducted by the Central Statistical Agency of Ethiopia, the zone has a total population of 6,142,063 in an area of 4,208.64 square kilometers (1,624.96 sq. mil). There are fourteen hospitals in Zone, five private and nine public hospitals, 74 health centers, and 355 health posts. These public hospitals (one comprehensive specialized hospital and eight primary public hospitals) are Wolaita Sodo University Comprehensive Specialize Hospital (WSUCSH), Halale Primary Hospital, Bombe Primary Hospital, Bale Primary Hospital, Gesuba Primary Hospital, Boditi Primary Hospital, Bedessa Primary Hospital, Humbo Primary Hospital, and Bitena Primary Hospital. Wolaita Zonal Health Department data showed that there were 4,845 permanently employed health care professionals in these public hospitals. The data was collected from January 1–30, 2023.

### Study population

#### Source population

All healthcare professionals who were working in public hospitals of Wolaita Zone.

#### Study population

All healthcare professionals who were working in public hospitals in Wolaita Zone found during data collection period and fulfilled the inclusion criteria for quantitative study, and key informant healthcare professionals who agreed to take part in interview were included for qualitative study.

### Eligibility criteria

#### Inclusion criteria

Those healthcare professionals who were permanently employed, had work experience of at least six months, had a willingness to participate in the study, and were found during the data collection period were included in the quantitative study, while healthcare professionals with at least one year of experience and a willingness to be interviewed were included in the qualitative study.

#### Exclusion criteria

Those healthcare professionals who were temporarily employed and unable to participate in the study due to illness, annual leave, or maternity leave at the time of data collection were excluded from both the quantitative and qualitative studies.

### Sample size determination and sampling technique

**Fig. 1:**
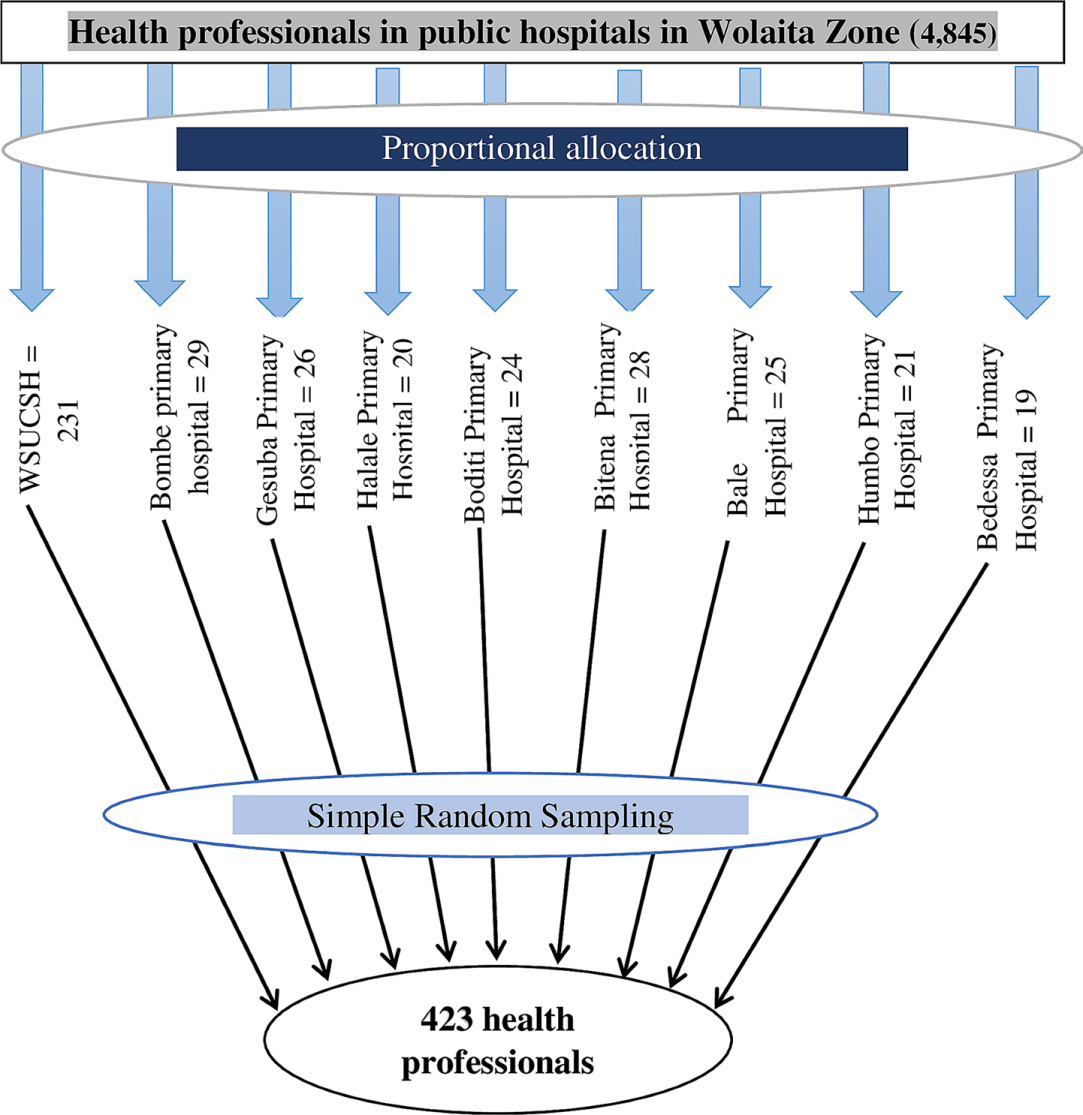
Schematic presentation of sampling procedure in public hospitals of the Wolaita Zone, Southern Ethiopia, 2022 (*n* = 423)

For quantitative study, sample size was determined by using s single population proportion formula considering the most recent proportion of 50.1% taken from a study conducted previously in Bale Zone, South Eastern Ethiopia [12] with a 5% margin of error and a 95% confidence level. After accounting for a 10% non-response rate, the final sample size was 423. All nine public hospitals in Wolaita Zone was included in the study and there were 4,845 permanently employed healthcare professionals working at these public hospitals during the study period. A proportional allocation was conducted for each hospital based on the actual number of healthcare professionals based on the following proportional allocation formula: nj = $\frac{n.Nj}{N}$ where: nj is the sample size in j hospital, n is estimated final sample size (*n* = 423), j is the total number of healthcare professionals in j hospital, and N is the total number of healthcare professionals in nine public hospitals in Wolaita Zone Hospitals of Wolaita Zone (*N* = 4,845) Finally, simple random sampling technique using a lottery method was used to select the healthcare professionals by using their salary payroll lists from human resource office of each public hospital as a sampling frame (Fig. 1).

By using a purposive sampling technique, the sample size for the qualitative study was determined. Nine representative healthcare professionals were chosen from public hospitals and took part in key informant interviews until content saturation.

### Data collection tool and technique

For the quantitative study, an English-language, pretested, structured, self-administered questionnaire was used to gather the data. The questionnaire was categorized into five parts and obtained from earlier research conducted in different areas [12, 14, 20]. The first part of the questionnaire contains the sociodemographic characteristics of the study healthcare professionals. The second part contains organizational-related factors affecting the practice of informed consent. Items in the third and fourth sections assessed the knowledge and attitude of respondents about informed consent, respectively. The final part contains items to assess the healthcare professionals’ practice of the informed consent process. Thirteen Likert scale questions (i.e. I obtain informed consent from patients for major surgical procedures or other treatments; I inform the patients why the proposed procedure will be performed; and I inform the patients about the presence or absence of alternative treatment options for the proposed treatment; I inform the patients about the type of anaesthesia to be used during surgery; I explain the benefits of the proposed procedure to the patient; I explain the risks or potential complications related to the proposed procedure to the patient; I explain a favorable environment to say no to the proposed procedure; I inform the patients about the consequences of refusing the proposed procedure; I provide counselling aids, including the recommended treatment, that assist in decision-making for the patients; I provide adequate time for patients to sign the informed consent form; I provide an opportunity to ask questions of the patients; I assess the competence of my patients to give consent to treatment or procedure; and I check that my patients understand the explanations I provided to them) were used assess the practice of informed consent. The collection of data was conducted by nine BSc-level healthcare professionals with four supervisors who participated in supervising the overall activities during the data collection period. In each hospital, data collectors were tasked with distributing and collecting the questionnaire from healthcare professionals. For the qualitative study, a qualitative, semi-structured, face-to-face individual key informant interview was conducted with nine healthcare professionals (one healthcare professional from each hospital). A semi-structured interview guide with open-ended questions developed for this study and reviewed by experts was used. During the interview, we have asked a variety of probing questions, like Tell me more about that. Would you repeat it? Would you elaborate more on it? What do you mean when you say…? and so on) according to the participants’ responses for clarity and an in-depth understanding of the phenomenon under investigation. The interview was done in Amharic language by the principal investigator, and all the conversation during key informant interview was recorded using an electronic voice recorder based on the interview guide developed. The healthcare professionals were asked about their experiences with and insights into informed consent.

### Study variables

#### Dependent variable

Informed consent practice.

#### Independent variables

Sociodemographic characteristics (age, sex, religion, ethnicity, language, marital status, educational status, experience, hospital level and monthly income).

Organizational factors affecting the practice of informed consent (working unit, adequate content of informed consent, training on informed consent, institutional policy or regulation, administrative support, lack of standard consent form, time constraint, workload).

Knowledge on informed consent.

Attitude on informed consent.

### Operational definition and measurements

#### Informed consent

is a person’s decision, given voluntarily, to agree to a healthcare treatment, procedure, or other intervention.

#### Informed consent practice

The process through which healthcare professionals give patients the necessary information to enable them to voluntarily and intelligently make decisions about their care. The healthcare professionals’ practice of informed consent was measured by thirteen Likert-scale questions having three points, namely “never,” “sometimes,” and “always,” with scores of 1, 2, and 3, respectively. Then the total score was dichotomized into two groups using the mean as the cut-off point. A score above or equal to the mean on the practice questions is considered good practice. A score below the mean on the practice questions is considered poor practice.

#### Knowledge towards informed consent

refers to healthcare professionals’ understanding of the ethical, legal, and procedural aspects of the informed consent process. To measure the knowledge of healthcare professionals on informed consent practice, ten questions with “yes,” “no,” and “I don’t know” options were utilized. Each item received a score of 1 for a valid response (i.e., yes) and 0 for an incorrect response (i.e., “no” and “I don’t know”). Using the mean score as the cut-off point, the total knowledge score was dichotomized into two groups. A score above or equal to the mean for the knowledge items indicates having good knowledge, and a score below the knowledge questions’ mean indicates poor knowledge.

#### Attitude towards informed consent

refers to their healthcare professionals’ individual beliefs, values, and dispositions regarding the importance, respect, and application of the informed consent process in healthcare settings. There are nine attitude items with a 5-point Likert types were used to measure the attitudes of healthcare professionals towards a proper informed consent process. Responses were given with scores of 1, 2, 3, 4, and 5 for strongly disagree, disagree, neutral, agree, and strongly agree. Using the mean score as the cut-off point, the participants’ attitudes were dichotomized into two groups. A score greater than or equal to the mean for the attitude items indicates a favorable attitude, while a score less than the mean for the attitude questions indicates an unfavorable attitude.

#### Data processing and analysis

For the quantitative study the collected data were entered into Statistical Package for the Social Sciences (SPSS) Version 26 for analysis. Descriptive statistics such as tables, graphs, frequencies, and percentages were used to describe the sample’s characteristics. The bivariable and multivariable logistic regression method was used to find the association between dependent and independent variables. All independent variables with a p-value less than 0.25 from the bivariable logistic regression model were entered into the multivariable logistic regression model. A significant association was obtained at an adjusted odds ratio (AOR) with a 95% confidence interval (CI) and p-value less than 0.05 for interpretation.

For the qualitative study, as soon as possible following the key informant interview, the voice recordings were transcribed word for word in Amharic before analysis, and the information was then translated into English. The translated data were then imported into OpenCode software for coding, and for this study, content thematic analysis was carried out, themes and subthemes were identified, and an expert in qualitative study from Wolaita Sodo University validated it. Finally, an original verbatim quotation from participants was also employed.

#### Data quality control

To assure the quality of the data for the quantitative study, one day of training was given to the data collectors on the data collection tool and how to conduct the collection. A pre-test of the questionnaire was conducted in Shone Primary Hospital, which is outside of the target hospitals, on 5% of the sample size a week before the actual data collection period, and necessary amendments were made such as to clarify unclear questions, correct typing errors, and clarify ambiguous words accordingly. Cronbach’s alpha was computed to assess the internal consistency of the Likert-scale questionnaire during the pretest for this study (0.792 for informed consent practice items, 0.823 for knowledge towards informed consent items, and 0.889 for attitude towards informed consent items). The process of data collection was supervised by the principal investigator. The principal investigator also checked the completeness, accuracy, and consistency of the collected data every day. The double data entry method was used by two data clerks, and the consistencies of the entered data were cross-checked by comparing the two separately entered data on SPSS. For the qualitative study, interviews were conducted in a quiet room when there was no hectic situation, and the data was transcribed and translated. The interview was discontinued after it was determined that data saturation had been achieved. The criteria for trustworthiness (credibility, reliability, confirmability, and transferability) were maintained throughout the investigation. Prior to translation, experts checked audio recordings and transcribed notes for accuracy and completeness.

## Results

This study aimed to examine the implementation of informed consent and identify the factors influencing its practice among healthcare professionals. The key findings indicate a markedly poor practice of informed consent. The following information provides in-depth insights into the outcomes of our study, focusing on the sociodemographic characteristics of healthcare professionals, the execution of informed consent, the knowledge and attitudes of healthcare professionals regarding informed consent, as well as the factors associated with informed consent practice.

### Sociodemographic characteristics of healthcare professionals

A total of 399 healthcare professionals were participated in this study giving a response rate of 94.3%. Among the respondents, 223 (55.9%) were males, and the mean age of the respondents was 31 years with a standard deviation of 6.7. Protestant religion followers made up 170 (32.6%) of the participants, while Wolaita ethnicity made up 243 (60.9%). In terms of marital status, 280 (70.2%) of the respondents were married, and half (49.4%) of the study participants had a bachelor’s degree or higher. Two hundred fifty-six (64.2%) of the healthcare professionals were working in primary hospitals, while the rest were working in comprehensive specialized hospitals. In terms of work experience, 186 (46.6%) of healthcare professionals have at least 5 years of experience, and the average monthly salary of respondents was 5668.00 birr with a standard deviation of 1540.00 (Table 1).

**Table 1:** Sociodemographic characteristics of healthcare professionals working in public hospitals in Wolaita Zone, Southern Ethiopia, January, 2023 (n=399)

Variable Name	Category	Frequency(n)	Percentage (%)
Age in years	20–29 years	156	39.1
	30–39 years	161	40.4
	≥ 40 years	82	20.5
Gender	Male	223	55.9
	Female	176	44.1
Religion	Orthodox	155	38.8
	Protestant	170	42.6
	Muslim	51	12.8
	Others	23	5.8
Ethnicity	Wolaita	243	60.9
	Amhara	48	12.0
	Dawro	44	11.1
	Sidama	38	9.5
	Others	26	6.5
Marital status	Single	119	29.8
	Married	280	70.2
Educational status	Diploma	139	34.8
	First degree	197	49.4
	Second degree and above	63	15.8
Experience	< 2 years	102	25.6
	2–5 years	111	27.8
	> 5 years	186	46.6
Monthly salary	≤ 4085 ETB	70	17.5
	4086–5294 ETB	79	19.8
	≥ 5295 ETB	250	62.7
Hospital level	Comprehensive specialized	143	35.8
	Primary	256	64.2

ETB: Ethiopian Birr

### Practice of informed consent among healthcare professionals

Out of 399 healthcare professionals who participated in the study and worked in public hospitals in Wolaita Zone, 212 (53.1%) [95% CI of 48.98–57.92%] had good practice of informed consent.

### Knowledge of healthcare professionals towards informed consent

**Fig. 2:**
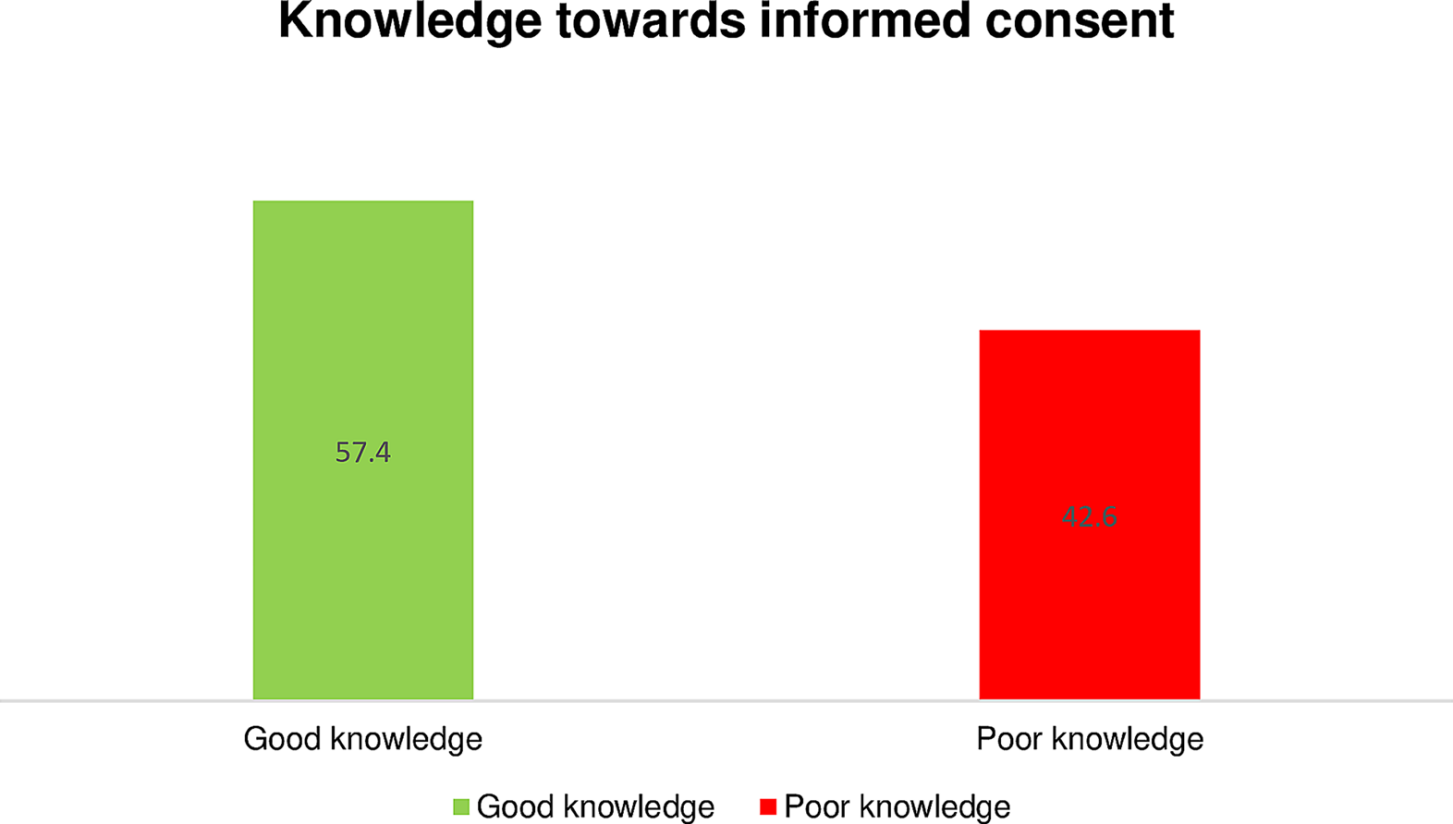
Knowledge towards informed consent among healthcare professionals working in public hospitals in Wolaita Zone, Southern Ethiopia, January, 2023 (*n* = 399)

The good knowledge towards informed consent among healthcare professionals in public hospitals in Wolaita Zone was 229 (57.4%), whereas 170 (42.6%) of the healthcare professionals had poor knowledge towards informed consent (Fig. 2). Among the respondents, 299 (74.9%) said that they knew the informed consent process, and 253 (63.4%) said that they knew the requirements for valid consent. From the healthcare professionals, 331 (82.9%) responded that informed consent is a legally regulated process in healthcare organizations, and 291 (72.9%) of the them responded that they know the components of the informed consent process should be disclosed to patients.

### Attitude of healthcare professionals towards informed consent

**Fig. 3:**
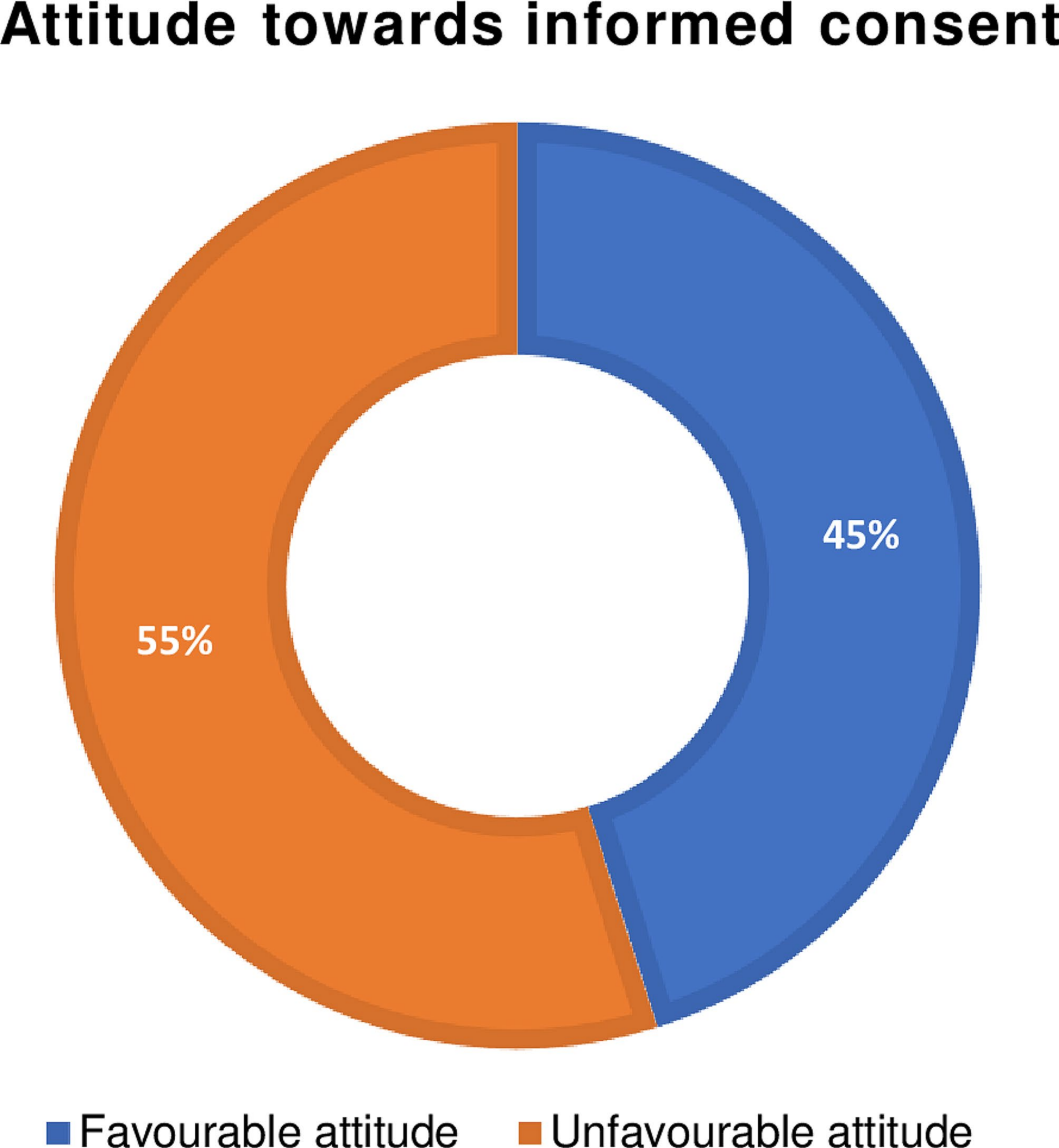
Attitude towards informed consent among healthcare professionals working in public hospitals in Wolaita Zone, Southern Ethiopia, January, 2023 (*n* = 399)

According to this study, 180 (45.1%) of the respondents had a favorable attitude towards the practice of informed consent (Fig. 3). Two hundred eight-one (70.4%) of respondents strongly agreed that the preconditions for obtaining consent are patient competence and voluntariness, while 191 (47.3%) remained neutral on the idea that decision-making about proposed treatment or procedure does not belong to the health care provider. Similarly, 332 (83.2%) of the respondents agreed that all the risks of a proposed treatment need to be explained to the patient. About 200 (50.1%) disagreed that it is important to repeat the explanation if the patient demands it.

### Organizational factors affecting practice informed consent

Among the study respondents, 208 (52.1%) responded that they have gotten in-service training on informed consent, and 110 (27.6%) responded that the average time spent for obtaining consent form ranges from 11 to 20 min. Similarly, 226 (56.6%) of the healthcare professionals said that there was availability of institutional policy/regulation in their hospital about informed consent process, and 46.4 (46.4%) reported that there got administrative support from hospital managers on issues related with informed consent. However, 232 (58.1%) of the respondents reported that there was no adequate informed consent content in their hospital, and 164 (41.1%) said that give a care for 6–10 patients per shift (Table 2).

**Table 2:** Organizational factors influencing practice of informed consent among healthcare professionals working in public hospitals in wolaita zone, Southern Ethiopia, January, 2023 (n=399)

Factors		Frequency(n)	Percentage (%)
In-service training	Yes	208	52.1
	No	191	47.9
Working unit	Medical ward	86	21.6
	Surgical ward	83	20.8
	Pediatrics ward	57	14.3
	Obs/Gyn ward	27	6.8
	Emergency ward	64	16.0
	ICU	36	9.0
	OPD	39	9.8
	Others	7	1.8
Time spent for consenting	< 5 min	68	17.0
	5–10 min	105	26.3
	11–20 min	110	27.6
	21–30 min	55	13.8
	> 30 min	61	15.3
Availability of institutional policy/regulation	Yes	226	56.6
	No	173	43.4
Administrative support	Yes	185	46.4
	No	214	53.6
Adequate informed consent content	Yes	167	41.9
	No	232	58.1
Number of patients cared per shift	< 5 patient	80	20.1
	6–10 Patient	164	41.1
	> 10 patient	155	38.8

ICU: In-Patient Department, OPD: Out-Patient Department, Oby/Gyn: Obstetrics and Gynecology

### Factors Associated with informed consent practice

In bivariable logistic regression, sex, educational status, experience, monthly income, hospital level, time spent for consenting, availability of institutional policy/regulation, administrative support, in-service training, and attitude toward informed consent were all candidates for multivariable logistic regression analysis (*p* < 0.25). In bivariable logistic regression, sex, hospital level, and in-service training were significantly associated with the practice of informed consent (*p* < 0.05). Male healthcare professionals were 1.03 times more likely to practice good informed consent than female healthcare professionals [AOR: 1.030 (95% CI: 0.011–0.072)]. Healthcare professionals working in comprehensive specialized hospitals were 4.78 times more likely to practice good informed consent than those who were working in primary hospitals [AOR: 4.775 (95% CI: 1.45–15.74)]. Healthcare professionals who had gotten in-service training were 1.16 times more likely to practice good informed consent than those who did not get in-service training [AOR: 1.161 (95% CI: 0.735–2.042)] (Table 3).

**Table 3:** Bivariable and multivariable binary logistic regression analysis on factors associated with practice of informed consent among healthcare professionals working in public hospitals in Wolaita Zone, Southern Ethiopia, January, 2023 (n=399)

Variables	Informed consent practice		COR	AOR (95% CI)	p-value
	Good	Poor			
Educational status					
Diploma	68	71	1	1	
BSc degree	106	91	0.63	1.142 (0.33–3.93)	0.833
Master and above	38	25	0.82	4.061 (0.89–18.47)	0.070
Sex					
Male	204	19	1.004	1.030(0.011–1.072	0.001*
Female	28	148	1	1	
Work experience					
< 2 years	32	26	1	1	
2–5 years	101	73	0.890	1.045 (0.25–4.42)	0.952
< 5 years	79	88	1.37	2.45 (1.99–5.56)	0.892
Monthly income					
≤ 4085 ETB	30	40	1	1	
4086–5294 ETB	45	34	0.567	1.569 (0.25–9.92)	0.632
≥ 5295 ETB	137	113	0.619	0.835 (0.16–4.29)	0.829
Hospital level					
CSH	84	59	0.702	4.775 (1.45–15.74)	0.010*
Primary Hospital	128	128	1	1	
Time spent for consenting					
< 5 min	36	32	0.754	0.931 (0.17–5.096)	0.934
5–10 mi	58	47	0.688	1.256 (0.26–6.06)	0.776
11–20 min	54	56	0.880	1.162 (0.23–5.79)	0.855
21–30 min	36	19	0.448	0.269 (0.04–1.75)	0.169
> 30 min	28	33	1	1	
Institutional policy					
Yes	110	116	1.52	3.01 (0.99–9.13)	0.052
No	102	71	1	1	
Administrative support					
Yes	105	80	0.76	2.620 (0.87–7.92)	0.088
No	107	107	1	1	
In-service training					
Yes	130	78	1.25	1.161 (0.735–2.042)	< 0.001*
No	129	62	1	1	
Attitude					
Favorable	166	14	0.022	0.559 (0.108–2.91)	0.489
Unfavorable	46	173	1	1	

*= significant association, COR: Crude Odds Ratio, AOR: Adjusted Odds Ratio

## Key informant interview result

Nine healthcare experts participated in a key informant interview. The participants are between the ages of 24 and 38. Using the software OpenCode 4.03, the data were organized into three themes based on the data gleaned from the key informant interview.

### Theme one: Factors related to hospital administration

Almost all healthcare professionals who participated in key informant interviews identified poor hospital administration as a factor influencing informed consent practice, such as a lack of appreciation, assessment, and on-the-job training. It has been reiterated that ongoing monitoring or evaluation is absent regarding the execution of informed consent in the hospitals. Moreover, there is a lack of cohesive cooperation with healthcare professionals who could serve as exemplary figures or possess a profound comprehension of informed consent. Additionally, there is a deficiency in both on-the-job and off-the-job training initiatives aimed at enriching the knowledge, mindset, and proficiency of healthcare professionals in the realm of informed consent practice.

A 28-year-old female clinical midwife said, *“I think there should be continuous assessment about the practice of informed consent so that the healthcare professionals can practice informed consent in their daily activities when dealing with patient care.”*

A 32-year-old male BSC nurse said the following: *What I think is a solution for this poor practice of informed consent is that first, health professionals should learn from each other. For example, there might be a professional who can be a role model for other staff, so others should learn from and take experience from him. The hospital management should also create on-the-job training opportunities to enhance the professional’s knowledge and attitude toward the practice of informed consent.*

A 27 years old male general nurse said *“…I haven’t ever heard about training about practice of informed consent. There are not training opportunities at all in our hospital. Basically, training is very important for professionals to improve knowledge and attitude on practice of informed consent.*

Another 38 years old male laboratory technician said: *“*…*The managers don’t acknowledge or appreciate what we are doing for the patients. Professionals are not motivated to give informed consent if the management bodies don’t provide for their requirements or show them that they care (at the very least, by saying things like “Thank you” or “Be strong” or something like). The outstanding professionals should be rewarded positively for what they are doing (like obtaining informed consent) by different criteria, such as CRC (caring, respectful, and compassionate).*

### Theme two: Factors related resource availability

Six out of nine healthcare professionals of key informant interview reported that there are limited resources such as adequate content of informed consent, lack of standard consent forms, and limited time. They also pointed that there is also high flow of patient with low number of staffs in some hospitals which leads to high workload among the staffs. The disproportion between the number of patients or clients seeking health services and the limited number of healthcare professionals proved to be a significant impediment to the proper implementation of informed consent. Likewise, the unavailability of essential resources, such as standardized consent forms, posed a crucial challenge in ensuring the effective utilization of informed consent within healthcare facilities.

A 25 years old male nurse professionals explained *“…I never thought about obtaining informed consent from patients because our hospital has an excessive patient flow, and the nurse-to-patient ratio is unbalanced. For instance, I would be expected to take care of over 15 patients in a single shift. I don’t have enough time to obtain consent from my patients since I have a lot of work to do and care for them.*

A 30 years old female public health professionals said, *“… a shortage of resources prohibits our hospital from having an informed consent form. The allocation of resources, even on informed consent forms, clearly differs between our primary hospitals and those of general, specialized comprehensive, or tertiary hospitals.*

### Theme three: Factors related with healthcare professionals

The majority of healthcare professionals in key informant interviews stated that the major barriers to informed consent practice were lack of experience, inadequate understanding, unfavorable attitudes, and equating care with rewards and incentives. Experienced healthcare professionals play a vital role in delivering high-quality healthcare services. Nevertheless, the dearth of experienced personnel has emerged as a significant obstacle to the effective implementation of informed consent. Furthermore, the lack of motivation stemming from inadequate support for exceptional performers, low salary levels, delayed or absent overtime payments from hospital management, and the absence of affirmative actions tailored specifically for female healthcare workers have all had a detrimental impact on the implementation of informed consent within their respective hospitals.

A 26 years old medical doctor commented this; *“Health care is a mind work, so there will be nothing to prevent implementation of informed consent, but there are health professionals who attach and see activities such as informed consent with benefits and incentives, for example, with duty and other incentives or with salary compliance, so there is dissatisfaction with payments and benefits and professionals become negligent in practicing it. But, since we are professionals, we should not associate such activities with benefits, since it is our mandate to act.”*

Another 37 years old female nurse who has 12 years of working experience explained that *“…I believe that lack of experience is the most common barrier to healthcare professional’s inadequate informed consent practices. Actually, if the hospital has its own rules and regulations, the professionals may abide by them and follow them, but rules and regulations alone are insufficient for a professional to practice informed consent; you also need to gain experience in order to improve your knowledge and attitude toward the practice, which lowers your negligence rate.*

A 25-year-old female BSc midwifery professional said, “…*In our hospital, the female healthcare workforce takes up the lion’s share of total human resources. But when you see it at this level, there is no affirmative action for female professionals. There are no rewards or other motivations for outstanding female workers. So, if you are not motivated, you may have no desire to do specific jobs in your profession such as obtaining informed consent from your patients. Sometimes, patients might not have equal feelings for male and female healthcare professionals. That means they are inclined towards male healthcare professionals and have more respect for them. This might be because our community gives more priority to males than females, and this problem is still existing. This leads to demotivation, and sometimes, I get upset about my profession.*

A 33 years old female diploma nurse who has 11 years’ experience said *“…If the situation is not life-threatening, such as major surgery, I do not believe it is a serious problem to not obtain informed consent. For instance, transplantation may be a dangerous situation, so the nurses or other professionals should take getting informed consent very seriously, whereas taking vital signs is not required for all patients or clients since there are no hazards involved for the patient.*

## Discussion

This study aimed to discover the nursing practices of informed consent of healthcare professional working in public hospitals in the Wolaita Zone. It also tried to identify factors that were associated with the practice of informed consent. The practice of informed consent among healthcare professionals working in public hospitals of Wolaita Zone, Southern Ethiopia, was 53.1%. This finding was in line with the study conducted in the Bale Zone, Ethiopia (50.1%) [12]. This might be due to similarities in socioeconomic characteristics, the health policy of the country, and the educational system of healthcare professionals.

However, this finding was lower than the findings of the study in Italy (71.0%) [17]. This discrepancy might be due to the differences in study settings (the study in Italy was conducted in six large teaching hospitals, whereas this study was conducted in one comprehensive specialized and eight primary hospitals). Additional reasons might be differences in study participants, study periods, sample sizes, and differences in educational policies, economies, and infrastructure between the countries. Similarly, it might be due to differences in knowledge and attitudes of healthcare professionals towards the practice of informed consent. Differences in health systems between the countries might also be the reason for this disparity. For example, Breaches in the quality of informed consent practice are frequently described in developing countries due to lower levels of formal education, differences in health structure, and limited access to healthcare supplies than in developed countries [24].

The finding of this study was higher than the study conducted in Uganda (48.8%) [18]. The possible reason for this difference might be differences in study time gaps, sample sizes, study participants (the above study were conducted only on nurses, while this study was conducted on overall healthcare professionals), and sociodemographic characteristics of the study participants, as well as differences in data collection methods. The other plausible reason for this disparity might be due to cultural, structural, or healthcare system differences. For instance, researchers from the Maya Health Alliance of Guatemala and the USA found that differences in cultural communication outcomes affect the mistaken impressions produced by unconscious bias and inquiries made by practitioners, their interpretation of patients’ responses, and their degree of empathy may all be influenced by unconscious cultural biases. Consequently, this affects patient assessments and care [25]. Similarly, a study conducted in Vietnam showed that differences in social and cultural factors can shape the practices of informed consent and result in a fragmented understanding of information [26].

The first significant factor associated with the practice of informed consent was the gender of healthcare professionals. Female healthcare professionals were 3% likely to have good practice informed consent than male healthcare professionals. This finding was consistent with the studies conducted in Southwestern Ethiopia, and Italy [12, 17]. The possible explanation for this consistency might be due to the fact that the number of male healthcare professionals is higher than the number of female healthcare professionals. Another reason could be that females have a greater family and societal formal and informal workload outside of their hospital duties. Similarly, this might be because the communication skills of male and female healthcare professionals may differ. It is conceivable that male healthcare professionals have effective communication skills and can explain to patients the specifics of informed consent. This may lead to higher compliance and understanding from their patients when it comes to informed consent procedures. Another reason for this might be that male healthcare workers may interact or communicate with patients in specific ways that lead to fruitful conversations about informed consent. For instance, patients may be more likely to believe and heed the advice of male medical professionals, which could result in better rates of informed consent procedure adherence. This finding has been supported by the qualitative component of our research, which aligns with factors about healthcare professionals or personal circumstances. Healthcare professionals who participated in key informant interviews revealed that patient preference is more for male healthcare professionals, and a lack of motivation and rewards for their outstanding performance from the organization might have a negative effect on the practice of informed consent. Consequently, the best course of action to address these issues is to raise patient and community awareness while also boosting the motivation and ambition of female healthcare professionals through rewards and incentives.

The level of hospital is also significantly associated with the good practice of informed consent. Those healthcare professionals working in comprehensive, specialized hospitals were 4.78 times more likely to have a good practice of informed consent than those who were working in primary hospitals. This might be due to the fact that in-service training opportunities and the availability of adequate informed consent content might be more available in comprehensive specialized hospitals than primary hospitals. Most of the healthcare professionals with an educational status of master’s degree and above are also working in comprehensive specialized hospitals. There might also be more in-service training opportunities than in primary hospitals. This finding was also supported by the qualitative part of the study, which goes in line with resource availability and hospital administration challenges. Healthcare professionals in our key informant interview mentioned that the differences in administrative capacity and resource availability between primary and comprehensive specialized hospitals have a great impact on the practice of informed consent. They also pointed out that higher-level hospitals have better resource availability and better training opportunities with local, national, and international organizations than primary hospitals, and this might have a positive effect on informed consent practice.

Another variable significantly associated with the good practice of informed consent was in-service training. Healthcare professionals who had not received in-service training were 4% less likely to practice informed consent compared to those who had received in-service training. This finding was in line with the studies conducted in the Bale Zone, Ethiopia, and the Democratic Republic of the Congo [12, 20]. The reason for this similarity might be the fact that increased opportunities for in-service training among healthcare professionals increase their knowledge and attitude towards informed consent practice and also increase the value healthcare professionals place on obtaining informed consent from the patient. Training has a positive impact on the practice of informed consent, enhancing knowledge among healthcare professionals, improving communication skills, increasing awareness of fundamental ethical considerations, establishing standardized procedures for obtaining informed consent, encouraging a patient-centered approach to care, familiarizing healthcare professionals with regulatory requirements, contributing to ongoing quality improvement efforts, and others. In contrast, if there are no or only limited opportunities for training, informed consent becomes less frequently practiced among healthcare workers. This finding was supported by the qualitative part of the study, which aligns with factors related to hospital administration. Healthcare professionals who participated in the key informant interview explained that the main issue with the poor implementation of informed consent in our study area is a lack of training, and the hospital administration did not organize various training opportunities about informed consent to enhance their knowledge, attitude, and practice. Therefore, training will enhance healthcare professionals’ willingness to practice informed consent.

### Implications of the study

The findings of this study could provide several distinct theoretical and practical implications for healthcare professionals, hospitals, and policymakers. It helps to fill the research gap and also provides valuable insights that can improve the practice of informed consent in Ethiopia and in Africa at large. It alerts hospital management and other stakeholders to become more knowledgeable about the practice of informed consent and the factors affecting it. Informed consent has benefits by creating trusting relationships between healthcare professionals and patients, and it has the benefit of increasing patient satisfaction with the care given in hospitals. It is very important to improve it by applying different evidence-based strategies to maximize the practice of nurses on informed consent.

### Conclusion and recommendation

This study assessed the practice of informed consent among healthcare professionals and the factors associated with it. More than half of the healthcare professionals in public hospitals in Wolaita Zone had good practices for informed consent. This could be considered low because this finding implied that almost half of the healthcare professionals had poor informed consent practices. Being male, working in comprehensive specialized hospital and having in-service training had significant associations with informed consent practice. It is recommended that hospital managers and health policymakers plan and intervene with different strategies organizing appropriate training opportunities in collaboration with the Ethiopian Federal Ministry of Health and regional health bureau or non-governmental organizations, applying supervision and feedback mechanisms, and providing necessary materials and supplies of informed consent, to improve the practice of informed consent among healthcare professionals. Future researchers are also recommended to conduct longitudinal research to confirm a definitive cause-and-effect relationship between the practice of informed consent and the independent variables.

### Strength of the study

All public hospitals in Wolaita Zone were included, which makes it more representative and generalizable, and variables that were overlooked in the previous studies were included to address the effects of these variables on the practice of informed consent.

### Limitation of the study

The cross-sectional nature of the study design does not show a cause-and-effect relationship between the dependent and independent variables. Additionally, since the data for the quantitative study were collected through a self-administered questionnaire, it might be subjected to response bias from the respondents, and recall bias may also exist among participants, which could have decreased the observed associations.

### Electronic supplementary material

Below is the link to the electronic supplementary material.

Supplementary Material 1

## Data Availability

The datasets used and/or analyzed during the current study are available from the corresponding author on reasonable request.

## Abbreviations

AOR: Adjusted Odds Ratio
CI: Confidence Interval
COR: Crude Odds Ratio
CSH: Comprehensive Specialized Hospital
ETB: Ethiopian Birr
SPSS: Statistical Package for the Social Sciences
USA: United States of America
WSUCSH: Wolaita Sodo University Comprehensive Specialized Hospital
