# Supplementary material for: Informed consent practice and associated factors among healthcare professionals in public hospitals of Southern Ethiopia, 2023: a mixed-method study
Source: BMC Nurs. 2024 Jan 30;23:77. doi: 10.1186/s12912-024-01748-9 (PMC10826175; doi:10.1186/s12912-024-01748-9)
Supplement: Supplementary file 1 — Supplementary Material 1 [file 12912_2024_1748_MOESM1_ESM.docx]

**Guide for Key Informant Interview**

**Greetings**: We are currently doing a research to assess informed consent practice and associated factors among healthcare professionals in Wolaita Zone, Southern Ethiopia public hospitals. We are going to ask you some questions that are very important for the healthcare professionals, patients and policy makers among others make to improve the patient safety and quality of health service concerning an informed consent. Your response to this interview will remain confidential and anonymous.

**Thank you for your participation in the interview.**

**QUESTIONS**

1. Would you tell me about your hospital and working unit/ward-------------------------------?
2. How important is to discuss the informed consent practice as part of patient safety practice?
3. How informed consent is implemented through the process in your hospital?
4. What is the reason of practicing of informed consent?
5. What are the factors that hinders the practice of informed consent practice?
6. Why patients are consented to participate in decision making about proposed treatments and procedures?
